# Supplementary material for: Knowledge, attitudes and practices on household solid waste management and associated factors in Gelemso town, Ethiopia
Source: PLoS One. 2023 Feb 10;18(2):e0278181. doi: 10.1371/journal.pone.0278181 (PMC9916587; doi:10.1371/journal.pone.0278181)
Supplement: S1 File — (DOCX) [file pone.0278181.s001.docx]

**Supplementary file**

**S1 File Survey questionnaire (English version)**

ADDIS ABABA UNIVERSITY

College of Natural and Computational Sciences

Department of Biology

Dear respondents, the aim of this questionnaire is prepared to gather relevant information about the **knowledge, attitude and practice on household solid waste management in Gelemso town, Ethiopia**. So your genuine and correct responses will contribute for my effective work. Your information will be kept confidential and not used for other purpose and hence you are personally not affected. For this reason you are kindly provide the correct information for the following questions.

GENERAL INFORMATION

- Do not write your name.
- Please put”√” mark in boxes.
- Circle the correct letter from each questions.
- Provide relevant information for open ended questions on space provide.
- Fill the questionnaire without discussing with your friends.

**SECTION I. QUESTIONNAIRES FOR HOUSEHOLDS’ RESPONDENTS**the information obtained through this questionnaire will be used only for academic Purpose and could not have any effect on the respondents’ privacy.

**Part I. Respondents’ of Personal Information**

1. Kebele; A, 01 🞏 B, 02 🞏

2. Sex: A, Male 🞏 B, Female 🞏

3. Age in year: __________

4. Education level: A, No formal education (illiterate) 🞏 B, Primary school 🞏

C, Secondary school D,Diploma🞏 F, Bachelor’degree and above 🞏

5. Duration of stay in the town: A, < 2 years 🞏 B, 2-5years 🞏 C, 6-10 Years 🞏 D, Above 10 years 🞏

6. Family size: A, 1-3 B, 4-6 C, 7-9 D, above 10

7. Average household’s monthly income in birr: A, Less than 1000 🞏 B, 1000-1500 🞏 C, 1501-3000 🞏 D, 3001-5000 🞏 E, above 500O 🞏

8. Occupation: A, Government employee 🞏 B, NGO 🞏 C, Self-employed🞏 D, employed in Private sectors 🞏 E, Other (specify) __________

**Part II. Knowledge of Households towards Solid Waste Management**.

Please, choose one of the alternatives by put “√” in the given tables.

| No | Questions | Alternatives | |
| --- | --- | --- | --- |
|  |  | A | B |
| 1 | Does solid waste is source of pollution for the environment? |  |  |
| 2 | Burning of solid wastes causes health risks (eg. bronchitis and asthma) |  |  |
| 3 | Waste papers, plastic bags, a piece of metal, wood and cloths are recyclable? |  |  |
| 4 | Do you consider solid waste is being a wealth? |  |  |
| 5 | Can solid waste be sorted and sold for recycling companies? |  |  |
| 6 | Compost or organic fertilizer can be prepared from solid waste? |  |  |
| 7 | The amount of solid waste can be reduced by reusing at household level? |  |  |
| 8 | Illegal damping of solid waste causes diarrhea, typhoid, and cholera? |  |  |
| 9 | Sorting of solid waste at home level helps for SWM? |  |  |
| 10 | Improper dumping of solid wastes can eventually lead to pollution of rivers, lakes and wells? |  |  |

Alternatives: A= Yes B= No

**Part III. Attitude of Households towards Solid Waste management**

Please, choose one of the alternative scales by put “√” in the given tables.

|  |  | Alternative | | | |
| --- | --- | --- | --- | --- | --- |
| No | Statements | A | B | C | D |
| 1 | Solid waste is anything without value. |  |  |  |  |
| 2 | Solid waste is one of the environmental problems that need an immediate attention |  |  |  |  |
| 3 | Solid wastes can be reduced, reused and recycled (3Rs) |  |  |  |  |
| 4 | Every household should have responsibility for the proper collection and disposal of solid wastes |  |  |  |  |
| 5 | Proper solid waste disposal is the responsibility of everyone |  |  |  |  |
| 6 | Proper SWM is important for creating healthy environment |  |  |  |  |
| 7 | SWM is a burning issue in the town |  |  |  |  |
| 8 | The city government should conduct regular supervision and control on illegal dumping of solid waste in the town |  |  |  |  |
| 9 | Selling plastic waste for recycling is the best way to manage solid wastes |  |  |  |  |

Alternative Scales: A=Strongly Agree B=Agree C=Disagree D=Strongly Disagree

Part **IV. Households’ Practice in Solid Waste Management.**

| Please, answer by circling one of the alternatives for the following questions: |  |  |  |  |
| --- | --- | --- | --- | --- |

1. What are the typical components of your household solid wastes? A, Khat leftover and food waste B, Plastic, beverage cans, glass and bottle C, Metal, leather, and textile D, Paper and cardboard E, Others
2. How do you get rid of solid wastes from home A, Dump in backyard with sacs

B, Dumped along roadsides or in gully C, Dumped in disposal site D, Buried in the soil

1. How are the content and volume of solid waste changes from time to time in the town?

A, Increased B, Decreased C, No change

1. The frequency of burning your household solid waste will be: A, Daily B, once in two or three days C, Weekly D, Monthly
2. How is the collection and disposal service of solid waste provided by the municipality? A, Good B, Satisfactory C, Poor
3. Do you separate solid wastes before disposal? A, Yes B, No
4. Do you practice reduce, reuse and recycle strategy for SW or not use 3R?A, Reduce by compost preparation (R-1) B, Reuse (R-2) C, Selling for recycling business (Korale and Lewache*) (3R) D, Burn (Not use 3R)
5. Are there adequate solid waste landfills or dumping sites in the town? A, Yes B, No
6. Do you have access to door-to-door waste collection service? A, Yes B, No
7. How do you transport solid waste to the nearby container? A, by member of the households B, Paying for micro enterprise C, Private waste collectors D, Other means

**Section II. Focus group discussion (FGDs)**

ADDIS ABABA UNIVERSITY

College of Natural and Computational Sciences

Department of Biology

Dear members of the focus group discussion, the aim of this discussion is prepared to gather relevant information about the “**knowledge, attitude and practice on household solid waste management in Gelemso town, Ethiopia”**. So your genuine discussion will contribute a great value for my effective work. Our discussion will be kept confidential and not used for other purpose and hence you are personally not affected. We hope that our discussion will be fruitful and relevant to this particular work and following are points (questions) useful for our discussion.

1. How is the volume and content of solid wastes generated from households year to year in the town?

2. Could you please describe how you collect solid wastes from households? 3. How often do you collect solid wastes from the households in designated area?

4. Is there any sorting of solid wastes at household levels?

5. Do you think that sorting of solid waste at home level could significantly important?

6. Could you please describe where you take the solid wastes you are collected from the households?

7. Do you agree that the municipality has done enough public awareness on solid waste management?

8. What challenges do you faced when you are collecting and transporting solid waste from the households?

**Section III.. Key informant interview**

ADDIS ABABA UNIVERSITY

College of Natural and Computational Sciences

Department ofBiology

Dear members of the key informant, the aim of this interview is prepared to gather relevant information about the “**knowledge, attitude and practice on household solid waste management in Gelemso town, Ethiopia”**. So your genuine interview will contribute a great value for my effective work. Our interview will be kept confidential and not used for other purpose and hence you are personally not affected.For this reason you are kindly provide the correct information for the following questions.

1. Do you agree that the municipality has done enough public awareness on solid waste management?­­­­­­­­­­­­­________________________________________________________________________________________________________________________________________________________
2. What are the major constituents of solid wastes in your town?

________________________________________________________________________________________________________________________________________________________

3. How are the content and the volume of solid waste changes from time to time in the town?

________________________________________________________________________________________________________________________________________________________

4. Are there adequate solid waste landfills or dump sites in the town?

________________________________________________________________________________________________________________________________________________________

5. Have you a plan to encourage the households to reuse and recycle of solid wastes in your town?

____________________________________________________________________________________________________________________________________________________________

6. Are the households accepting the decision made by your office on solid waste management in the town?

________________________________________________________________________________________________________________________________________________________

7. What are some of the challenges in dealing with solid waste management?

____________________________________________________________________________________________________________________________________________________________

8. What measures your office has taken to solve solid waste management challenges?

____________________________________________________________________________________________________________________________________________________________
