# Supplementary material for: Knowledge, attitudes and practices on household solid waste management and associated factors in Gelemso town, Ethiopia
Source: PLoS One. 2023 Feb 10;18(2):e0278181. doi: 10.1371/journal.pone.0278181 (PMC9916587; doi:10.1371/journal.pone.0278181)
Supplement: S2 File — (PDF) [file pone.0278181.s002.pdf]

Request for Permission to Publish Content under CC-BY License

Dear Rights Holder or Representative,

I have submitted a paper for publication in a PLOS journal, and wish to include the content listed below in the paper. I'm hereby requesting your (or your company's or institution's) permission to include the content in my paper. Please note that all PLOS journals are published under a Creative Commons Attribution License (CC BY), which allows for unrestricted use and distribution, even commercial, as long as attribution is given to the creator or rights holder of the content. See https://creativecommons.org/licenses/by/4.0/.

To grant me permission to use the content in my PLOS paper, please fill in the information below and then scan the completed form and send it to me at my email address.

Thank you.

My name:

Fitsum Tigu

My email address:

fitsum.tigu@aau.edu.et
fitsumtigu@yahoo.com

Description of the content which I'm seeking permission to use (citation and/or title, and pasted screen shot, if applicable):

Figure 1 Collection of solid wastes by Korale and Lewache for recycling (Source: Field observation by researcher). Left to right: plastics, beverage cans, glass and bottles; metals, corrugated iron sheets; and textiles and shoes.

Link to the Content:

* * *

On behalf of myself or the rights holder, I hereby grant the permission sought herein.

Signature of Party Granting Permission:

Fitsum Tigu, [signature]

Date:

Jan. 05, 2023

Printed Name and Title:

Figure 1 Collection of solid wastes by Korale and Lewache for recycling (Source: Field observation by researcher). Left to right: plastics, beverage cans, glass and bottles; metals, corrugated iron sheets; and textiles and shoes.
