# Supplementary material for: Knowledge, attitudes and practices on household solid waste management and associated factors in Gelemso town, Ethiopia
Source: PLoS One. 2023 Feb 10;18(2):e0278181. doi: 10.1371/journal.pone.0278181 (PMC9916587; doi:10.1371/journal.pone.0278181)
Supplement: Attachment — Submitted filename: Response to reviewers.doc [file pone.0278181.s003.doc]

06 May 2022

We gratefully acknowledged the academic editor and reviewer for their valuable comments to our manuscript. The following are point-by-point responses to the academic editor and reviewer comments for the manuscript number: **PONE-D-22-02551.**

Reviewer #1:

**Q#1**: Title does not look meaningful and attractive. It may be changed and result oriented.

**Answer**: *Modified as ‘Knowledge, attitude and practice on household solid waste management in Gelemso town, Ethiopia’.*

**Q#2**: Write up is very poor (view comment in attachment).

**Answer**: *We thank the reviewer for valuable comments. We have made significant improvement on the write up of the manuscript according to the comments. The revised parts are indicated in the track change form.*

***NB****: We confirm that there is no attached documents/comments with the editorial manager website/by email. Thus, we have done the correction by ourselves.*

**Q#3**: Material and method section should be improved accordingly.

**Answer**: *Again we appreciate the reviewer for valuable points. We had addressed the issue properly and indicated in track change form.*

**Q#4**: Results do not support the title. Interpretation of results is not clear.

**Answer**: *The authors also acknowledged the reviewer for critical observation. Following the comments, we have corrected the points which are not clear for the readers, some points lacking the right interpretations are corrected. By now you will find it clear and legible. Again the corrections are indicated in revised manuscript with track change.*

**Q#5**: Discussion section must be re-written before re-submission.

**Answer**: *We found points which are really required clarification, thus we have done the revisions properly. Plus to this, we made a general revision to the discussion section and all the mistakes and unclear parts are significantly improved and the changes are indicated by track change form.*

**Q6#. Conclusion section must be linked with the quantified results. General statements commonly mislead the readers.**

***Answer***: *Thank you very much for the comment raised by the reviewer. We have carefully corrected. The corrected points are indicated in the marked version with track change.*

**Q7#. English language may be improved. There are lot of language and grammar mistakes.**

***Answer***: *Thank you very much for the comment raised by the reviewer. We have thoroughly corrected the English issues of the manuscript and significantly improved the manuscript content accordingly. The corrected points are indicated in the marked version with track change.*
