# Supplementary material for: Knowledge, attitudes and practices on household solid waste management and associated factors in Gelemso town, Ethiopia
Source: PLoS One. 2023 Feb 10;18(2):e0278181. doi: 10.1371/journal.pone.0278181 (PMC9916587; doi:10.1371/journal.pone.0278181)
Supplement: Attachment — Submitted filename: Response to reviewers.doc [file pone.0278181.s004.doc]

25 October 2022

We are very much grateful to the academic editor who managed this manuscript again and also for the reviewers for their valuable comments to our manuscript. The following are point-by-point responses to reviewer comments for the manuscript number: **PONE-D-22-02551R1.**

Reviewer #2:

First of all, I would like to thank the second reviewer, who reviewed this manuscript and provided us the most valuable comments and suggestions. We received all the comments positively and frankly specking the manuscript is now well enriched.

**Q#1: Introduction**

**General comment:**

I found the Introduction incomplete and not well structured. You fail to introduce the knowledge gap your article is coming to fill.

**Answer**: *The whole introduction section is now modified according to the comments.*

When you focalize on Gelemso, the paragraph is disconnected from the first part. Moreover, you point to institutions as the main source of SW, but then you focus your research on households. Also, you do not include Khat consumption questions in your survey (or at least this is not presented in the Results analysis).

**Answer**: *The specific paragraph as well as the focus area of the research has been modified following the comments.*

You said, “Although there are limited SWM facilities in the town, most of the time the solid waste have been indiscriminately disposed along roadsides and into open areas and hence the current status of the SWM problem in the town has reached a critical stage.” But both facts may be correlated (as your results show, by the way). And then you state: “This shows that the people's overall understanding about solid waste and SWM practices in the town are not well established.” However, this is a hypothesis and not a statement.

**Answer**: *We accept the comments and modified the sentence accordingly.*

Specific comments:

Two first phrases of the 2nd paragraph should be unified.

**Answer**: *The comment is corrected*

**Q#2**: Methods

Specific comments:

In section 2.1, a description of the waste management system of Gelemso is needed. What kind of facilities people have access to, to dispose waste? Waste pickers are active in Gelemso? Have the Municipality a public waste collection service? Of what kind? How many containers are available? Who pick up waste from containers and where it is waste finally disposed?

**Answer**: *A paragraph explaining the whole waste management system of the Gelemso municipality including kind of facilities, waste pickers, waste collectors, the presence and number of shared container, damping sites and how the collected wastes are finally damped to the damping site are included in the “Method section” under sub section 2.1.*

In section 2.2, You should explain from which list you made the systematic random sampling. Do you have a list of all the households? Selected households they all accepted to participate in the study?

**Answer**: *The whole lists of the households were obtained from municipality office, and the section was made from the lists. The numbers of actual household who completed the questionnaire from the total sample size (396) were 390 (98.5% response rate). The following comments are now included and corrected in the revised manuscript.*

A satellite image of the town with the identification of the selected households would be a plus.

**Answer**: *We have included map of study area in supplementary file.*

In 2.3, the structure of the paragraph describing definitions should be reformulated because it is not understandable.

**Answer**: *Corrected as you suggested*

You skip from 2.4 to 2.7

**Answer**: *Corrected as you suggested*

Q#3: Results (General comment: all the data should be available)

Section 3.1: information presented in this section is not used in the Discussion section. Either you use it in section 4, or you remove it from section 3.

**Answer**: *Now included in the discussion part.*

Section 3.2: You say, “About 96% of the households gave the correct response regarding solid waste as the major pollutant of the environment”. Why can you affirm that SW is the main environmental pollution source?

**Answer**: *Now modified in the revised version.*

Many questions are not well formulated:

Does solid waste pollute the environment? --> It depends on how it is managed

Do you know solid waste is being a wealth? --> You give the answer in the question. "Do you consider..." "Do you think" "Do you perceive..."

The amount of solid waste can be reduced by reusing at household level? --> It is an obvious question

Sorting of solid waste at home level helps for SWM? --> It depends on how the system is structured and you did not gave the reader any elements to know that.

**Answer**: *Some of the questions including the mentioned questions were modified in revised version.*

Section 3.3: You give % of waste composition. But waste characterization studies have specific methodologies, and it seems you do not follow any of them. You should remove Figure 1 and all mentions of % of types of waste from your manuscript.

**Answer**: *All types of waste composition results were removed from the manuscript.*

You say, “However, the majority (79.5%) of the households disposed of their solid waste illegally, either in the backyard with sacs or along the roadsides and in gully (Table 4).” But maybe they expected a municipal kerbside collection. That’s why the Solid Waste Management system description is so important.

You say: “The majority (89.2%) of the households reported the absence of adequate solid waste landfills or damping sites in the city and thus had practiced an improper SWM (Table 4)”. It seems the problem is not people are not well informed but the absence of infrastructure!

**Answer**: *Descriptions of the solid waste management system in Gelemso town were described briefly in the revised manuscript. I hope the question is now understandable for the readers.*

3.4 In the questions you included: “How do you get rid of solid wastes from home”. Do you consider the possibility of people giving different destinations to recycling waste and non-recycling waste?

**Answer**: *Yes.*

Q#4: Discussion

First paragraph can be part of the Introduction.

**Answer**: *The paragraph was deleted from the discussion part according to the suggestion.*

After reading the discussion, it is still not clear why improper disposal is occurring in Gelemso.

**Answer**: *Now the discussion part is modified to why improper sold waste disposal is occurred in the study site.*

Q#5: Conclusion: Should be modified accordingly.

**Answer**: *The conclusion part has modified according to the comment in the revised manuscript.*
